# A Federated Online Search Tool for Biospecimens (Sample Locator): Usability Study

**DOI:** 10.2196/17739

**Published:** 2020-08-18

**Authors:** Christina Schüttler, Verena Huth, Magdaléna von Jagwitz-Biegnitz, Martin Lablans, Hans-Ulrich Prokosch, Lena Griebel

**Affiliations:** 1 Chair of Medical Informatics Friedrich-Alexander-Universität Erlangen-Nürnberg Erlangen Germany; 2 German Biobank Node Charité – Universitätsmedizin Berlin Berlin Germany; 3 Federated Information Systems German Cancer Research Center Heidelberg Germany; 4 University Medical Center Mannheim Mannheim Germany

**Keywords:** software tools, biological specimen banks, user interface, evaluation, research

## Abstract

**Background:**

The German Biobank Alliance (GBA) aims to establish a cross-site biobank network. For this endeavor, the so-called Sample Locator, a federated search tool for biospecimens and related data, has been developed, forming the heart of its information technology (IT) infrastructure.

**Objective:**

To ensure the sustainable use of such a tool, we included researchers as participants in an end user–based usability evaluation.

**Methods:**

To develop a prototype ready for evaluation, we needed input from GBA IT experts. Thus, we conducted a 2-day workshop with 8 GBA IT team members. The focus was on the respective steps of a user-centered design process. With the acquired knowledge, the participants designed low-fidelity mock-ups. The main ideas of these mock-ups were discussed, extracted, and summarized into a comprehensive prototype using Microsoft PowerPoint. Furthermore, we created a questionnaire concerning the usability of the prototype, including the System Usability Scale (SUS), questions on negative and positive aspects, and typical tasks to be fulfilled with the tool. Subsequently, the prototype was pretested on the basis of this questionnaire with researchers who have a biobank background. Based on this preliminary work, the usability analysis was ultimately carried out with researchers and the results were evaluated.

**Results:**

Altogether, 27 researchers familiar with sample requests evaluated the prototype. The analysis of the feedback certified a good usability, given that the Sample Locator prototype was seen as intuitive and user-friendly by 74% (20/27) of the participants. The total SUS score by the 25 persons that completed the questionnaire was 80.4, indicating good system usability. Still, the evaluation provided useful advice on optimization potential (eg, offering a help function).

**Conclusions:**

The findings of this usability analysis indicate that the considerations regarding a user-friendly application that have been made in the development process so far strongly coincide with the perception of the study participants. Nevertheless, it was important to engage prospective end users to ensure that the previous development is going in the desired direction and that the Sample Locator will be used in the future. The user comments and suggestions for improvement will be considered in upcoming iterations for refinement.

## Introduction

To align with the overarching goal of improving patient care by strengthening medical research, increased efforts have recently been made to support the secondary use of data generated in the treatment context [1-4]. In Germany, initiatives such as the Medical Informatics Initiative [5] or the German Biobank Alliance (GBA) [6] have emerged to establish an appropriate infrastructure for this endeavor. Coordinated by the German Biobank Node (GBN), the GBA has taken up the challenge of creating a cross-location biobank network to support biospecimen-based research projects. To this end, it is essential not only to ensure the quality of biosamples and associated data, but also to provide tools to improve the sample request process. It is currently customary for biosamples to be requested directly at a biobank location or via a mailing list. The request for fitting samples is usually carried out using a paper form. In addition, the negotiation regarding the sample distribution usually takes place by telephone or via email. GBA strives to bundle these heterogeneous steps into a single information technology (IT) application and thus align the individual process steps of the biobanks. The resulting harmonization should enhance the sample request for the researcher by providing access to the biosamples available in the biobanks via a single point of contact by means of one inquiry request. For this purpose, the so-called Sample Locator forms the core of the federated search for biospecimens and associated data [7,8]. The Sample Locator is intended to enable researchers to send a request to all connected biobanks via a central web application. As a first step, the researcher can check the potentially available number of samples across all locations in a feasibility query. After a positive response, the user can log in and access further functions. This includes the detailed breakdown of the number of samples per biobank, the management of queries, and the establishment of a direct electronic contact to the relevant biobanks. Crucial factors for sustainable use include not only the technical aspects, but also the user-friendliness of such a tool. In order to ensure usability and meet users’ needs, it is essential to involve end users in the development process. The reliance on the user-centered design (UCD) process has meanwhile proven itself in the development of IT applications in the medical (research) field [9]. A key aspect in the medical context is the avoidance of treatment errors due to lack of usability [10]. Even though the Sample Locator is not intended for application in the treatment context, a missing user-oriented approach can lead to the rejection of the tool. Therefore, its acceptance by the end users plays an influential role in our considerations.

The main objective of this paper is consequently to describe the evaluation of a midfidelity prototype in terms of its fitness for use. For the purpose of comprehensiveness, the paper also includes the necessary preparatory work, which covers the development of the prototype with the required functions and all steps of the request process. Since the focus of our work was to create a user-friendly search interface for the Sample Locator, we tested and assessed the usability of the prototype by end users, researchers familiar with sample requests. The resulting feedback will support the final tool development. An end user–based usability evaluation was used to ensure that the end users’ opinions on the Sample Locator was taken into account in potential further development steps.

## Methods

### Preliminary Work

A rough sketch with the required functions of the query process served as a starting point for the development of an interactive prototype. We first examined the search interfaces of already existing tools for a similar scope of application in order to get a clearer picture of how such a search tool could look in its final version. Moreover, to elaborate the sketch towards a prototype ready for evaluation, the input of GBA IT experts was needed. Thus, we planned and conducted a 2-day workshop with 8 GBA IT members, 7 men and 1 woman. Three researchers with expertise in usability moderated the workshop, in which individual steps of a UCD process were pursued in 2 groups of 4 people each [11]. First, the participants discussed the typical context of use of the Sample Locator, resulting in the design of 2 hypothetical users (hereafter referred to as personas) representing potential end users. Second, the participants defined 2 example interaction designs. For this purpose, the groups were each assigned a specific use case to describe a typical usage sequence to be executed with the tool. The use cases were derived from real requests from researchers to biobanks, which were previously collected for a requirements analysis to determine the scope of performance of the Sample Locator. The use cases (adapted from the German version) can be found in Multimedia Appendix 1. With the acquired knowledge, the participants designed low-fidelity mock-ups using Balsamiq (Balsamiq Studios) [12]. The main ideas of these mock-ups were discussed, extracted, and summarized into a comprehensive midfidelity prototype. It was modeled using Microsoft PowerPoint (Microsoft Corp) [13]. The individual slides were linked together with hyperlinks, achieving an interactive navigation through the prototypical tool. Regarding the presentation of the user interface, the website layout of GBN [14] served as a template to harmonize the look with GBN's corporate design. Subsequently, the first draft of the prototype was pretested by 3 male researchers with a biobank background who are affiliated with GBA. In total, 3 versions of the Sample Locator prototype were created in the course of this work. Following the iterative nature of the design process, the versions are based on each other. The second version, which the questionnaire described below refers to, was used for the usability tests. This in turn resulted in the third and final prototype, which was enriched by feedback from the evaluation.

### Study Design

Based on this preliminary work, the evaluation study was carried out. It was conducted as a usability analysis with female and male researchers aged between 18 and 67 years who work in a scientific institution in Germany. Prior to the start of the study, approval from the ethics committee of the Charité – Universitätsmedizin Berlin was obtained.

### Recruitment

The study plan envisaged the recruitment of between 30 and 50 respondents to the survey over a period of 6 weeks. With this number of participants, a detection of 95% to 98% of problems within an application can be expected [15]. In order to address suitable participants, one contact person per GBN partner biobank (n=13) was determined in advance to personally approach potential end users with information material. The study team chose this intermediary approach to ensure the anonymity of the participants. Except for the required activity in the research environment, there were no further inclusion or exclusion criteria. Subsequently, the identified participants received an email from the contact person with a link to the online survey tool, LamaPoll (Langner/Maibaum/Notev GbR) [16]. A first introductory page served to briefly inform the participants about the study and to provide the prototype via a download link. After agreeing to participate by clicking a consent button, the participant was forwarded to the study questionnaire.

### Instruments

The study questionnaire for the prototype evaluation consisted of 3 parts: (1) Sample Locator tasks and related questions on feasibility, (2) questions concerning the usability, and (3) general information.

In the first part, 4 tasks needed to be solved with the help of the provided prototype as a basis for subsequent questions. The first task was to search for samples of male patients with lung cancer. The others were to register to the Sample Locator, set project information, and refine the search query (lung metastasis samples plus excluding PAXgene-fixed tissue). The last task was to start negotiations with 2 selected biobanks. The tasks were designed to guide the test users through the prototypical system so they received insights into several possible functionalities. As with formulating the use cases for the workshop, these tasks were conceived based on the previously collected real inquiries to the biobanks. The first 6 questions enabled the researchers to evaluate the prototype’s general intuitiveness and comprehensibility using a 5-point Likert scale (1=strongly disagree, 3=neutral, 5=strongly agree). The following 10 open questions aimed to elicit opinions on content and appearance of the individual steps and further comments on the prototype.

The second part contained the System Usability Scale (SUS), a widely applied and validated score for the quantitative measurement of the usability of an IT application [17]. The wording was slightly adapted by changing “system” to “application” to facilitate the comprehension of the scale.

The third part collected information on age and computer-handling characteristics with 3 questions.

The final questionnaire (adapted from the German version) can be found in Multimedia Appendix 2.

### Data Analysis

The data from the pretest were not subjected to a sophisticated analysis due to the small amount of data, so they were immediately examined and implemented by a scientist. Two scientists documented and analyzed the collected data for the usability assessment. The applied data analysis methods include a quantitative evaluation for the SUS score, the calculation of the average rating and the standard deviations for all closed questions, and a descriptive qualitative content analysis for open questions. The result of the qualitative content analysis was a categorized list of comments and suggestions for improvement by topic, which was then prioritized according to its practicability. This means that changes to the user interface can be directly incorporated into the final prototype, while technical aspects must first be discussed with the developers.

## Results

### Results From Preliminary Work

**Figure 1:**
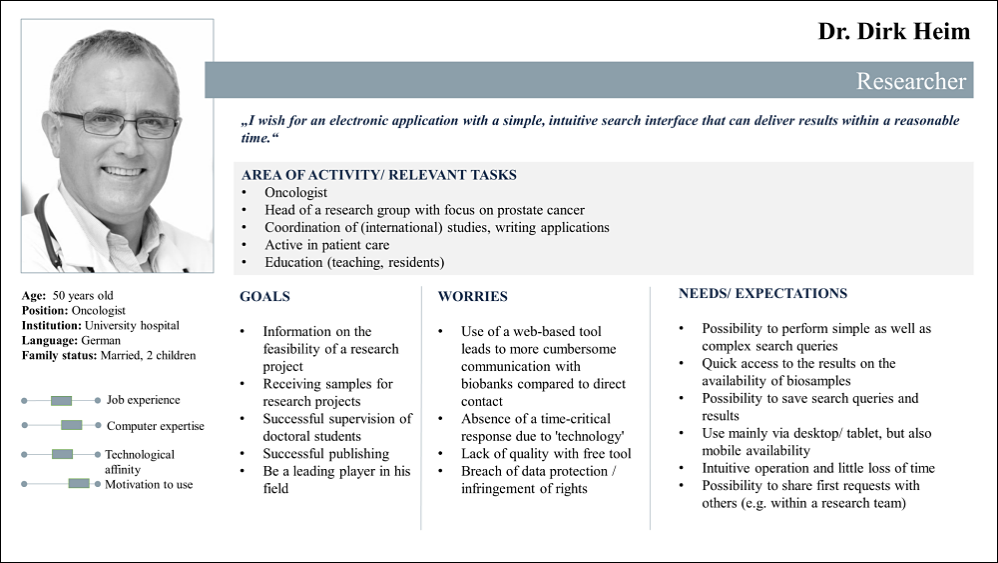
Persona of a researcher representing a future user of the Sample Locator.

The workshop laid the groundwork for the following usability analysis. Initially, the 2 groups participating in the workshop each created one persona of a researcher. Since the results overlapped to a high degree, the 2 outcomes were combined into 1 model persona (Figure 1) for a better overview throughout the course of the workshop. The next step—the development of the interaction designs—resulted in 2 sketched workflows of different application scenarios according to the use cases provided to the workshop participants. Here, the participants’ aim was to understand the essential steps of the process that were necessary for a successful trial of the respective use case and to visualize them in a flowchart (for the results, see Multimedia Appendix 3). Finally, the groups designed Sample Locator models by building on the previous steps, enabling the reproduction of the given use cases.

The first version of the prototype was consequently created considering the graphically sketched specifications of the functionalities and the mock-ups developed during the workshop (for some impressions, see Multimedia Appendix 4). The second version was a revision based on the feedback from biobank-based reviewers from the pretest. However, the revisions were mainly individual imprecise formulations that were corrected for better comprehensibility.

### Evaluation of the Prototype

A total of 27 participants complied with the call to respond to the survey and completed the questionnaire. Due to the recruitment setting, all participants were researchers who had already collaborated with biobanks and had experience in requesting samples. Thus, they represented typical end users of the Sample Locator. There were 6 persons aged 25 to 34 years, 18 persons aged 35 to 50 years, and 3 persons older than 50 years. Gender was not specifically surveyed in the study.

#### Sample Locator Tasks and Related Feasibility Questions

Most of the participants (20/27, 74%) agreed or fully agreed that the tasks were intuitively solvable using the prototype. Even more participants agreed or fully agreed that the display of the search process and the results were clearly arranged (22/27, 81%). The overall navigation was perceived as intuitive by approximately 74% (20/27) of the participants.

Participants particularly liked the absence of information overload and the prototype’s clear structure, which was “reduced to the essential.” Most testers confirmed this perception of clarity and structure for all of the process steps. Some mentioned that the mere prompt for International Classification of Diseases (ICD) codes for diagnoses was not usable “and it would be more convenient if the diagnoses...were behind the codes or if the list consisted entirely of plain text.” Additionally, one participant stated that the AND/OR/NOT conjunctions to refine the search criteria were not self-explanatory. Another critical voice requested that the search interface should allow defining dependencies between sample parameters (eg, whether the removal date is before or after therapy). The intuitive and simple handling of the tool was highlighted as positive. However, it was also suggested to offer a kind of “query builder, where [users] can still customize the generated query script as power user,” which allows more complex queries. Lastly, help functions were desired.

#### Questions About the Usability

Of the 27 participants, 25 (93%) completed the system usability questionnaire. The total SUS score of these 25 persons was 80.4, indicating good system usability.

The majority of the participants indicated that they would like to use the Sample Locator frequently (20/25, 80%). Additionally, most participants rated it easy to use (21/25, 84%). The complexity of the system was not seen as too high (21/25, 84% disagreed or fully disagreed that the complexity was too high). Regarding the need for technical support when using the Sample Locator, the results indicated a perceived high usability, as 23 of the 25 respondents (88%) disagreed or fully disagreed with the need for assistance.

The interface was seen as consistent (21/25, 84% disagreed or fully disagreed that the system was inconsistent). Most participants thought that their colleagues would easily learn to handle the Sample Locator (23/25, 92%), while 1 test person found the system hard to handle. Of the 25 participants, 21 indicated that they would feel confident using the Sample Locator. Nevertheless, 3 persons disagreed or fully disagreed with that. No participant indicated that he or she would need a lot of instruction before knowing how to use the Sample Locator.

#### General Information

All participants reported that they used a computer for fulfilling work tasks at least daily. Almost all persons (26/27, 96%) used a computer several times a day for their work. Of the 27 respondents, 10 persons (37%) indicated that they understood computers and computer technology very well, 15 persons (56%) understood computers well, and 2 (7%) self-assessed their understanding as sufficient.

### Implementation of the Feedback in the Final Prototype

**Figure 2:**
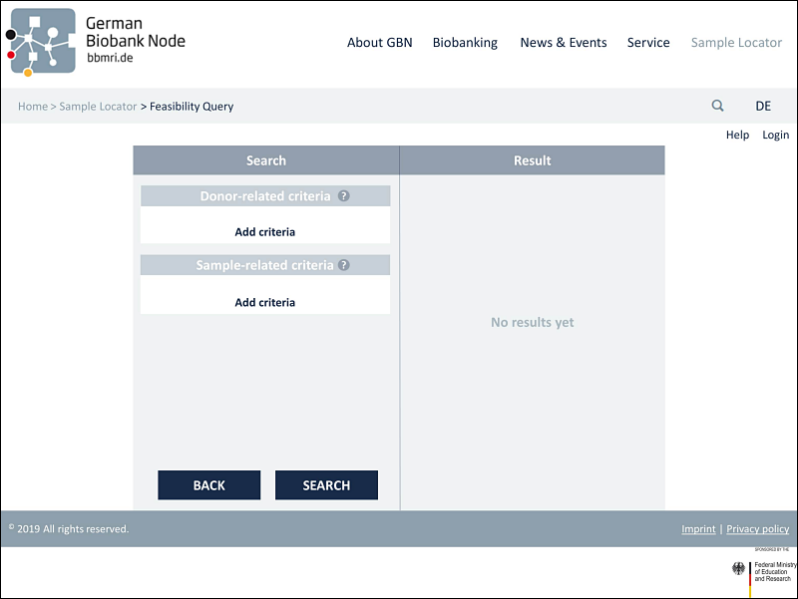
Initial search page with the basic functions of the prototype.

**Figure 3:**
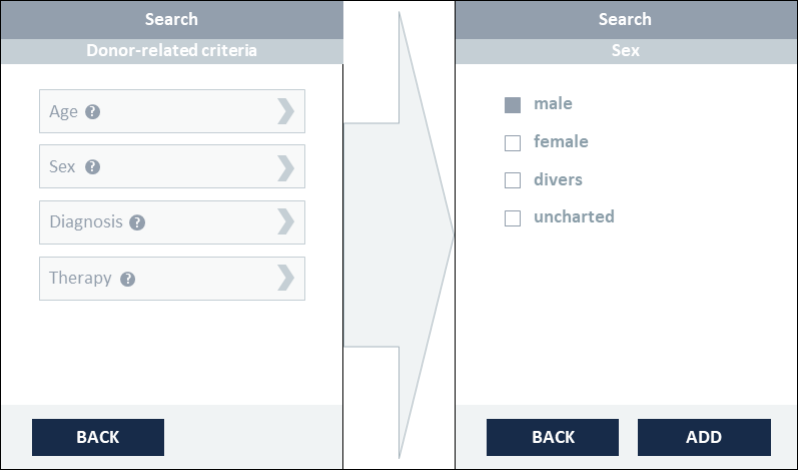
Illustration of the selection filters using the example of a patient with the sex parameter set to male.

**Figure 4:**
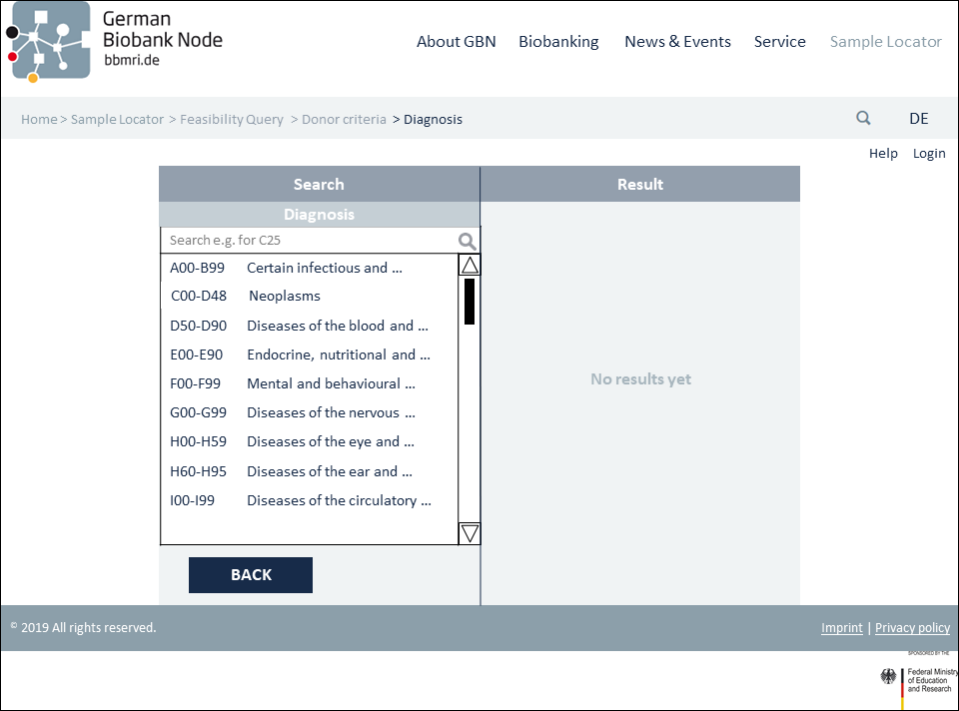
Continuation of the search for diagnoses by diagnosis classification and free text search.

**Figure 5:**
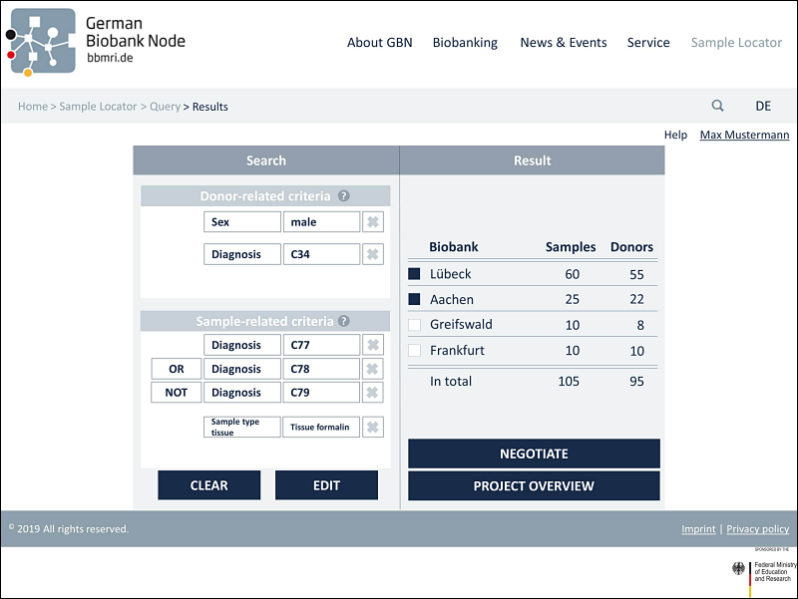
View of an example search query (left) with the corresponding detailed results per biobank (right).

The third and final prototype was modified in accordance with the responses of the usability analysis with researchers. Figure 2 illustrates the initial search page with the basic functions of the prototype’s third version. The search tool is divided into 2 sections. Search parameters can be selected in the left area of the screen. A distinction is made between sample-related criteria and donor-related criteria to obtain the most specific query possible. At this point, a help function has been added, as requested, which assists the user in finding the search parameters under the respective subheadings. Once the search criteria have been selected (eg, sex=male, as seen in Figure 3), the search can be performed by clicking the “Search” button. Here, a help function can now also be accessed to inform users about the format that can be searched for. A further implementation based on the test feedback when selecting the search parameters is the more detailed specification of the diagnostic codes. In addition to the simple code, the textual description has been added. In addition, the “direct entry of the ICD code (if already known) for input” is now possible via a free text field, as desired (Figure 4). The results will then be displayed in the right area of the screen. The feasibility query merely produces an aggregated number of potential samples and donor matches. As soon as the user has logged in to the tool in the next step, the allocation of suitable samples per biobank can be viewed (see Figure 5). In addition to an example query with detailed results for biobanks, Figure 5 also shows further functions. On the left “Search” side, the “Clear” button is used to completely restart a search, while the “Edit” button can be used to adjust the previous search. In order to have a better overview of which section you can currently interact with, a feature has been added that grays out and prevents editing within the inactive page. After the search has been carried out, one or more relevant biobanks can be selected on the right-hand side and contacted using the “Negotiate” button. The individual queries are saved and stored in a succeeding component. These can then be viewed and managed via the “Project Overview“ button. In addition, general design changes have been introduced (eg, adjusting the font size and color to improve readability). In view of the intended international use, the content was also translated into English, per recommendation. For extensive screenshots of the Sample Locator prototype, see Multimedia Appendix 5.

## Discussion

### Overview

The main goal of our work was to evaluate the usability of a federated search tool for biosamples and associated data, the Sample Locator. This paper also illustrates the work that was accomplished in advance of the usability analysis. However, the evaluation of the tool by the end users was of particular importance. The predominantly positive feedback of the usability analysis indicated an intuitive and user-friendly operability. The answers to the open questions provided useful advice on optimization potential.

### Comparison With Prior Work

In the area of medical research, especially with regard to cohort identification, several search tools are available. Commercial representatives include, among others, TriNetX [18], BC Platforms [19], and Clinerion [20]. Platforms or projects that make their work available as open source resources are i2b2 [21], tranSMART [22], and ConQuery [23]. The German Centre for Cardiovascular Research provides an in-house development for the feasibility query of biospecimens and data called the Feasibility Explorer [24]. The German Cancer Consortium (Deutsches Konsortium für Translationale Krebsforschung [DKTK]) has also implemented an in-house development (Searchbroker) as part of their comprehensive “bridgehead” architecture [8]. After reviewing these already existing tools, the deliberate decision was made to build on the DKTK codebase and their general architecture, but develop a customized, proprietary GBA Sample Locator solution. There were several reasons for this decision. Due to their developer power, industrial suppliers may have several advantages, particularly in the areas of speed and user interface design. However, especially when dealing with sensitive patient data, a solution that would be made available as an open source tool at the end of the project was preferred. In this case, all components can be developed, operated, and sustained by academic players, safeguarding the data sovereignty of biobanks. In this context, the Sample Locator, unlike tranSMART and the Feasibility Locator, follows a decentralized search approach. Consequently, the data remain locally at the biobank location, but can be queried centrally. Furthermore, we consider this open source approach to be a sensible way to ensure the continued existence of the Sample Locator beyond the end of the project [25]. In terms of further development, support, and bug fixing, the community resulting from this project (namely the Samply Community [26]) can continue to make a valuable contribution in the future. An additional functionality that distinguishes the Sample Locator from the search applications under review is that it limits the results visualization to the aggregated sample count from the whole GBA network for anonymous researchers. Only after a researcher’s authentication and log-in, the counts per biobank location are additionally displayed [6]. Further, after the search, the Sample Locator forwards the user directly to the Negotiator, a dedicated communication tool to help request material from relevant biobanks and negotiate the terms involved based on the actual planned research project. Thus, based on the considerations outlined above, none of the existing tools were suitable for direct application and deployment in GBA. However, the various approaches provided valuable input for GBA’s own search tool development in our incremental user-centered approach.

### Discussion of Results

#### Preliminary Work

As already mentioned, the procedure described in this paper for preparing and conducting the evaluation study of the Sample Locator was oriented towards the UCD process outlined in ISO 9241-210 [11]. However, due to time constraints inherent to the project and the fact that work had already begun on a development version to drive progress on the technical back end of the search tool, the focus was on the steps most relevant to our objective of providing a user-friendly application in avoidance of undue delay. This comprised the creation of a persona, the identification of an interaction design, the creation of an interactive prototype, and the evaluation of the prototype. In this shortened procedure, the thorough analysis of the user group in particular was neglected. Instead, our work was based on the project specification that the main users of the Sample Locator will primarily be researchers. The requirements of the users were derived from the real biobank queries that were previously collected. The persona and the interaction design not only provided an important input for the creation of the prototype but also served as orientation over the entire development process in order to visualize the user and the process of the product. The preparation of the prototype and its evaluation were aimed specifically at the engagement of those end users. This abbreviated approach still allows us to incorporate the users’ assessments in one of the following iteration steps in the further course of development.

Nonetheless, a limitation of our work might be that we used Microsoft PowerPoint as a tool for our prototype. Prior to creating the prototype, we also considered Balsamiq [12] and Axure [27] as alternative tools. Although these are dedicated tools for prototyping, we decided to use PowerPoint. On one hand, we wanted to have access to a tool that was as barrier free as possible. This applies to the licensing, handling, and distribution on our side as well as the use by the tester. PowerPoint is a widespread application that meets all our requirements in this respect, while the other tools would have required at least licensing and training to implement the project in the planned way. On the other hand, we did not strive to design a functioning, more realistic high-fidelity prototype. As a result, only the previously defined pathways resulting from the evaluation tasks were completely clickable. Although this presents a restricted interaction with the tool, this was sufficient for our purpose. The only constraint that became apparent in the course of implementation was the fact that there was not enough space to include more text, as the prototype was designed in typical PowerPoint slides. For example, ICD codes were not stored with textual descriptions, an aspect criticized by several test users. Apart from this limitation that prevented the test users from accurately experiencing the future tool’s fully featured performance, PowerPoint was a suitable tool for achieving our goal. However, we accepted this limitation, since the prototype should only represent the main functions and be the basis for further developments. With our approach, we were able to receive profound insight into the usability of the general design of the Sample Locator (eg, regarding button arrangements).

#### Evaluation of the Prototype

Al-Ageel et al [28] performed a literature review and found that bioinformatics tools should be engineered with usability in mind to allow the development of intuitive interactions in order to achieve learnability and ease of use in the interfaces. Usability problems such as inconsistent navigation may even prevent users from completing their tasks [29].

With our task-based approach, using a midfidelity prototype supplemented by the use of the SUS among bioinformatics experts, we found that the Sample Locator was intuitive and easy to use and that there was no information overload. Most participants wanted to use the tool frequently and felt that the interface was consistent.

Bolchini et al [29] identified usability issues that are potentially relevant for bioinformatics resources. One important usability issue with bioinformatics tools is the search function, which potentially leads to usability barriers [29]. For the Sample Locator, such usability problems were identified as well. For instance, the AND/OR/NOT conjunctions for refining the search criteria were not perceived as self-explanatory by 1 study participant. Another participant stated that the search interface should allow the definition of dependencies between different sample parameters (eg, whether the collection date of a biospecimen is before or after therapy). Some participants mentioned that it was not practical to use only ICD codes to search for diagnoses and that it would be more convenient if the diagnoses were behind the codes. From this, we can generally draw the lesson that we should present all information in an immediately understandable way so that even laypersons would comprehend it directly. However, the critical feedback from individual test persons should be considered as well, as this feedback could also be perceived as usability barriers for future end users.

The findings of the usability evaluation indicate that the prototype represented the main functions in a suitable manner. Nevertheless, several limitations have to be taken into account when interpreting the results.

First, the usability survey was performed at a relatively late development stage. A UCD process normally aims to include potential end users’ feedback in an early design phase [30]. Our approach was to assess the usability at a stage in which other attempts of designing the Sample Locator already existed. This unusual deferred approach was chosen because the first drafts of the Sample Locator focused more on technical possibilities, whereas our prototype’s aim was to measure potential end users’ opinions.

A further limitation inherent in our study is that we could only include 27 test users in our usability evaluation. This relatively small number of participants does not lead to statistically significant results, but merely provides insight into the participants’ opinions. However, our participants were experienced biobank users and thus could indeed provide reliable feedback. Moreover, it is not uncommon to use the SUS with a small number of participants. For example, Kersting and Weltermann [31] tested the usability of a software prototype supporting the management of multimorbid patients with 18 physicians. Likewise, Nielsen [32] stated that using 5 people in a study is sufficient for identifying almost all usability problems. We were able to gain valuable insights into test users’ expectations and opinions by using several open questions. Thus, we are confident that we have been able to identify the majority of usability problems of the prototypical Sample Locator.

By slightly changing the wording in one item of the SUS, we changed a validated scale. Therefore, the validity of the scale might not be a given anymore. Nevertheless, we have accepted this risk to ensure that the scale was understandable for our test users.

### Conclusion

The current lack of a suitable tool for a national search for biosamples prompted GBA to strive to close this gap. Although the initial focus was to provide a technically functioning development version at an early stage of the project, ensuring the user-friendliness of the tool should not be neglected in the process. To meet this demand, a usability analysis within the framework of a UCD process, which was also the basis for the accompanying preparatory work, was conducted. The findings of this usability analysis indicate that the considerations regarding a user-friendly application that have been made in the development process so far strongly coincide with the perception of the study participants. Despite this overlap, it was important to address the steps of the UCD process carried out in the context of this work and to engage prospective end users to ensure that the previous development was going in the desired direction and that the Sample Locator will be used in the future. Moreover, the users’ comments and suggestions for improvement that were received through the open questions will be considered in upcoming iterations to refine the Sample Locator. In this way, we are confident in the delivery of a final product that offers added technical value but is also intuitive for the user. These results will also have a positive impact on the aspired Europe-wide rollout. In Europe, the Biobanking and Biomolecular Resources Research Infrastructure – European Research Infrastructure Consortium Directory is currently commonly used as an online search tool, listing 720 European biobanks with their 1621 individual collections [33]. The Directory provides a detailed overview of the collections’ contents and enables users to contact a collection’s principal investigator. However, the Sample Locator would offer added value on a more granular level, enabling the search for specific samples and the contacting of the respective biobanks. While the biobank of Masaryk University in Brno is already connected, the further integration of European biobanks is planned.

## Appendix

Multimedia Appendix 1 Workshop use cases.

Multimedia Appendix 2 Questionaire evaluation study.

Multimedia Appendix 3 Interaction designs based on the provided use cases.

Multimedia Appendix 4 Preliminary work: sketched functionalities and workshop mock-ups.

Multimedia Appendix 5 Screenshots of the prototype.

## Abbreviations

DKTK: Deutsches Konsortium für Translationale Krebsforschung (German Cancer Consortium)
GBA: German Biobank Alliance
GBN: German Biobank Node
ICD: International Classification of Diseases
IT: information technology
SUS: System Usability Scale
UCD: user-centered design
