# Supplementary material for: A Federated Online Search Tool for Biospecimens (Sample Locator): Usability Study
Source: J Med Internet Res. 2020 Aug 18;22(8):e17739. doi: 10.2196/17739 (PMC7463387; doi:10.2196/17739)
Supplement: Multimedia Appendix 1 [file jmir_v22i8e17739_app1.pdf]

Multimedia Appendix 1 – Workshop use cases

Introduction:

Imagine you are a specialist in oncology and want to conduct a study on lung cancer.

Use case 1

You have heard of a web-based tool that allows you to centrally search for samples for your project and now you want to use it for the first time.

You want to submit a feasibility request for your study and search for biospecimen from male patients with lung carcinoids (C34.9).

After a successful search you would like to adapt your search again and additionally search for female patients and matching tumor tissue and whole blood.

Use case 2

You have already used the Sample Locator for a search query (search for tumor tissue of male patients with lung carcinogens [C34.9]) and now want to adjust some search parameters:

- Age at diagnosis: between 49 and 65 years
- Sex: male and female

The number of samples returned is sufficient for the study and now you want to contact the relevant biobanks.
