# Supplementary material for: A Federated Online Search Tool for Biospecimens (Sample Locator): Usability Study
Source: J Med Internet Res. 2020 Aug 18;22(8):e17739. doi: 10.2196/17739 (PMC7463387; doi:10.2196/17739)
Supplement: Multimedia Appendix 2 [file jmir_v22i8e17739_app2.pdf]

Alliance Partner
German Biobank Node
bbmri.de

FRIEDRICH-ALEXANDER UNIVERSITÄT ERLANGEN-NÜRNBERG
MEDIZINISCHE FAKULTÄT

Questionnaire for the evaluation study

"Online search for biosamples: Usability of the Sample Locator of the German Biobank Node"

Welcome to the presentation of the **Sample Locator**, an IT-supported tool for the cross-site search for biospecimen and related data.

This interactive prototype was developed by the **German Biobank Alliance** (GBA) under the supervision of the Erlangen site (Chair of Medical Informatics) and the GBA IT team. We would like to invite you as researchers and scientists to take part in our **20 to 30 minute survey** to evaluate the usability of the prototype. Participation in the survey is voluntary.

Please download the PowerPoint file (GBA prototype)! The online questionnaire contains tasks to be completed using the interactive prototype.

Link to the prototype

During the entire survey, no personal or personally identifiable data is collected, stored or processed except for your age (in the form of an age range). Your details are anonymous and will be treated confidentially. It is not possible to subsequently assign the answers of the participants. The results of the survey are used by GBA in aggregated form. You can cancel the processing of the online questionnaire at any time and without giving reasons.

If you have any **questions**, please contact Ms Christina Schüttler (christina.schuettler@fau.de).

Please click on "Agree" and then on "Continue" to participate in the survey.

☐ Agree

Part 1: Sample Locator tasks

Please try to solve the following tasks using the provided prototype. It is not a matter of whether you do something right or wrong - the tasks only serve as a basis for the subsequent survey on the usability of the prototype. Please also note that the prototype is mainly designed to solve the given tasks. Some paths may therefore not lead further, but only provide a brief insight into the sample locator.

Task 1)
You want to conduct a feasibility study to find out if enough samples are available for your research project. Please use the Sample Locator to search for samples from male patients with lung carcinoma (C34).

Task 2)
The aggregated result of the feasibility study with 846 samples from male patients with lung cancer is sufficient for your purposes. Now you would like to log on to the Sample Locator to find out which biobank offers how many samples. For this purpose, registration is necessary first. In addition, you will need to enter some details about your project in order to be able to manage your search queries and access the results of the individual biobanks. Please use the input already provided for these two steps.

Task 3)
You will now be directed to your search query with the detailed results per biobank. Now, you would like to refine the search query you have created. Please also search for samples with lung metastases (C78.0) and for matching tumor tissue (either tissue fixed to formalin or shock frozen, but no PAXgene-fixed tissue) for your sample collective.

Task 4)
After you have saved the search query, you can return to the result page and start a group chat with selected biobanks. To do this, select the two biobanks with the most samples (Lübeck and Aachen).

Questions relating to the handling of the tasks

1. I found the tasks to be intuitively solvable with the prototype provided.

strongly disagree 1	2	3	4	strongly agree 5
◌	◌	◌	◌	◌

2. I found the presentation of search and result to be clear.

strongly disagree 1	2	3	4	strongly agree 5
◌	◌	◌	◌	◌

3. I found the AND/OR/NOT combination of selected parameters to be comprehensible.

strongly disagree 1	2	3	4	strongly agree 5
◌	◌	◌	◌	◌

4. I found the project overview to be well structured and intuitive to use.

strongly disagree 1	2	3	4	strongly agree 5
◌	◌	◌	◌	◌

5. I found the sequence of the individual steps of the Sample Locator to be sensible and comprehensible.

strongly disagree 1	2	3	4	strongly agree 5
◌	◌	◌	◌	◌

6. I found the navigation through the Sample Locators intuitive.

strongly disagree 1	2	3	4	strongly agree 5
◌	◌	◌	◌	◌

7. Regarding the content and visual appearance of the feasibility study, I have noticed the following positive aspects:

8. Regarding the content and visual appearance of the feasibility study, I have noticed the following negative aspects:

Alliance Partner German Biobank Node bbmri.de

FRIEDRICH-ALEXANDER UNIVERSITÄT ERLANGEN-NÜRNBERG MEDIZINISCHE FAKULTÄT

9. Regarding the content and the visual appearance of the login and registration (login, individual registration, details of the project) I have noticed the following positive aspects:

10. Regarding the content and the visual appearance of the login and registration (login, individual registration, details of the project) I have noticed the following negative aspects:

11. Regarding the content and the visual appearance of the chat function I have noticed the following positive aspects:

12. Regarding the content and the visual appearance of the chat function I have noticed the following negative aspects:

13. On the prototype I have generally noticed the following positive aspects:

14. On the prototype I have generally noticed the following negative aspects:

15. I also noticed the following about the prototype:

16. For my search queries, the temporal proximity / temporal reference of individual criteria is of importance, e.g:

17. I would like to have the following additional functions for the Sample Locator:

Alliance Partner German Biobank Node bbmri.de

FRIEDRICH-ALEXANDER UNIVERSITÄT ERLANGEN-NÜRNBERG MEDIZINISCHE FAKULTÄT

Part 2: Questions concerning the usability of the Sample Locator

1. I could very well imagine using the application regularly.

strongly disagree 1	2	3	4	strongly agree 5
◌	◌	◌	◌	◌

2. I found the application unnecessarily complex.

strongly disagree 1	2	3	4	strongly agree 5
◌	◌	◌	◌	◌

3. I found the application easy to use.

strongly disagree 1	2	3	4	strongly agree 5
◌	◌	◌	◌	◌

4. I believe that I would need technical support to use the application.

strongly disagree 1	2	3	4	strongly agree 5
◌	◌	◌	◌	◌

5. I found that the various functions of this application were well integrated.

strongly disagree 1	2	3	4	strongly agree 5
◌	◌	◌	◌	◌

6. I think that the application contains too many inconsistencies.

strongly disagree 1	2	3	4	strongly agree 5
◌	◌	◌	◌	◌

7. I can imagine that most of my colleagues quickly learn how to use this application.

strongly disagree 1	2	3	4	strongly agree 5
◌	◌	◌	◌	◌

8. I found the application very cumbersome to use.

strongly disagree 1	2	3	4	strongly agree 5
◌	◌	◌	◌	◌

9. I would feel very confident in using the application.

strongly disagree 1	2	3	4	strongly agree 5
◌	◌	◌	◌	◌

10. I would have to learn a lot before I could work with the application.

strongly disagree 1	2	3	4	strongly agree 5
◌	◌	◌	◌	◌

Part 3: General information

1. What age group do you belong to?

☐ 18 to 24 years
☐ 25 to 34 years
☐ 35 to 50 years
☐ Over 50 years

2. How often do you use a computer to carry out your work tasks?

☐ Several times a day
☐ Daily
☐ Several times a week
☐ Weekly
☐ More rarely
☐ Never

3. Please assess how well you understand computers and computer technology.

☐ Very good
☐ Good
☐ Satisfying
☐ Sufficent
☐ Inadequate
