# Supplementary material for: A Federated Online Search Tool for Biospecimens (Sample Locator): Usability Study
Source: J Med Internet Res. 2020 Aug 18;22(8):e17739. doi: 10.2196/17739 (PMC7463387; doi:10.2196/17739)

Multimedia Appendix 3 – Interaction designs based on the provided use cases

Interaction design prepared for use case 1:

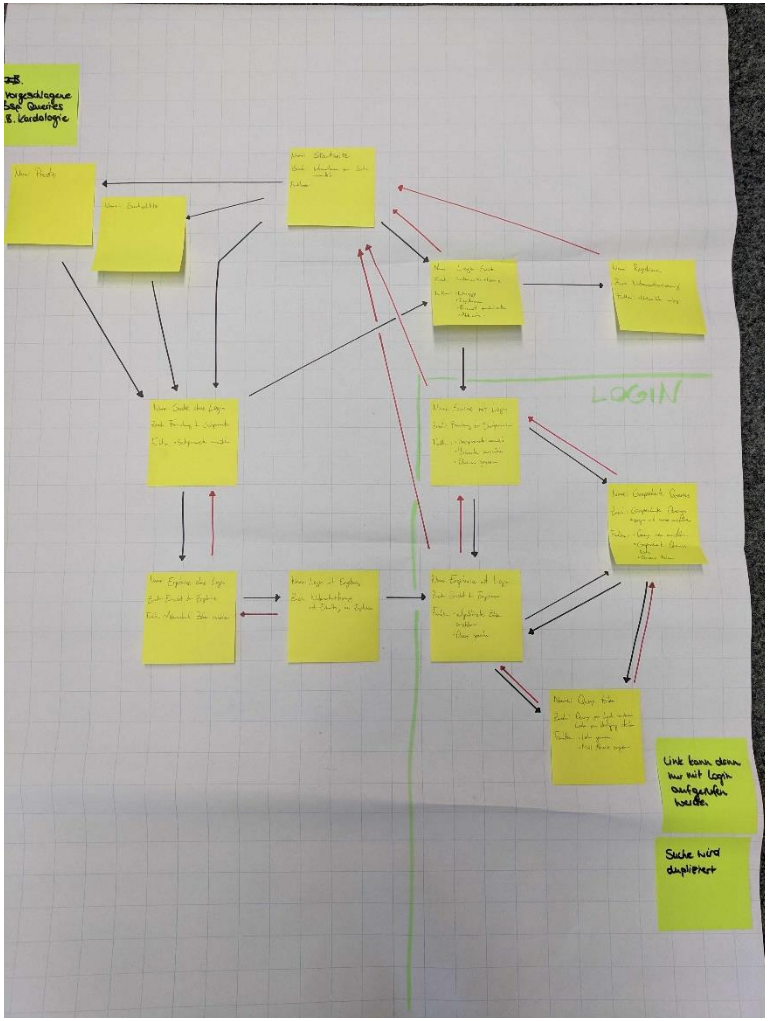

Interaction design prepared for use case 2:

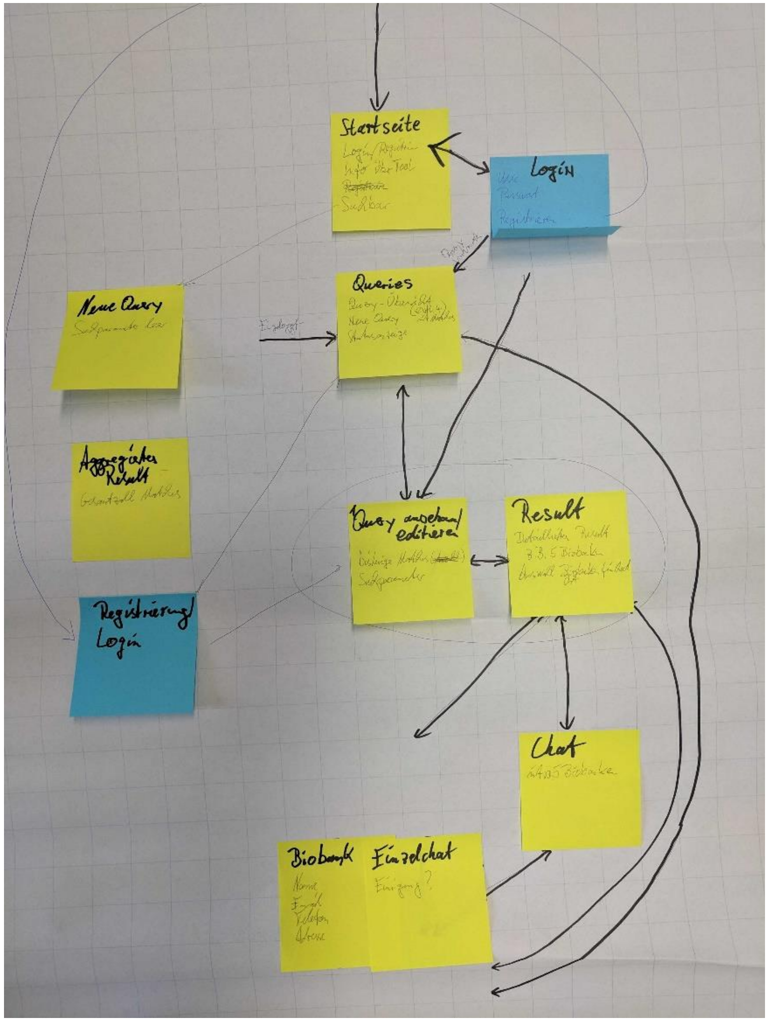

Supplement: Multimedia Appendix 3 [file jmir_v22i8e17739_app3.pdf]
