# Supplementary material for: A Federated Online Search Tool for Biospecimens (Sample Locator): Usability Study
Source: J Med Internet Res. 2020 Aug 18;22(8):e17739. doi: 10.2196/17739 (PMC7463387; doi:10.2196/17739)

Multimedia Appendix 4 – Preliminary Work: Sketched functionalities and workshop mockups

A. Sketched functionalities

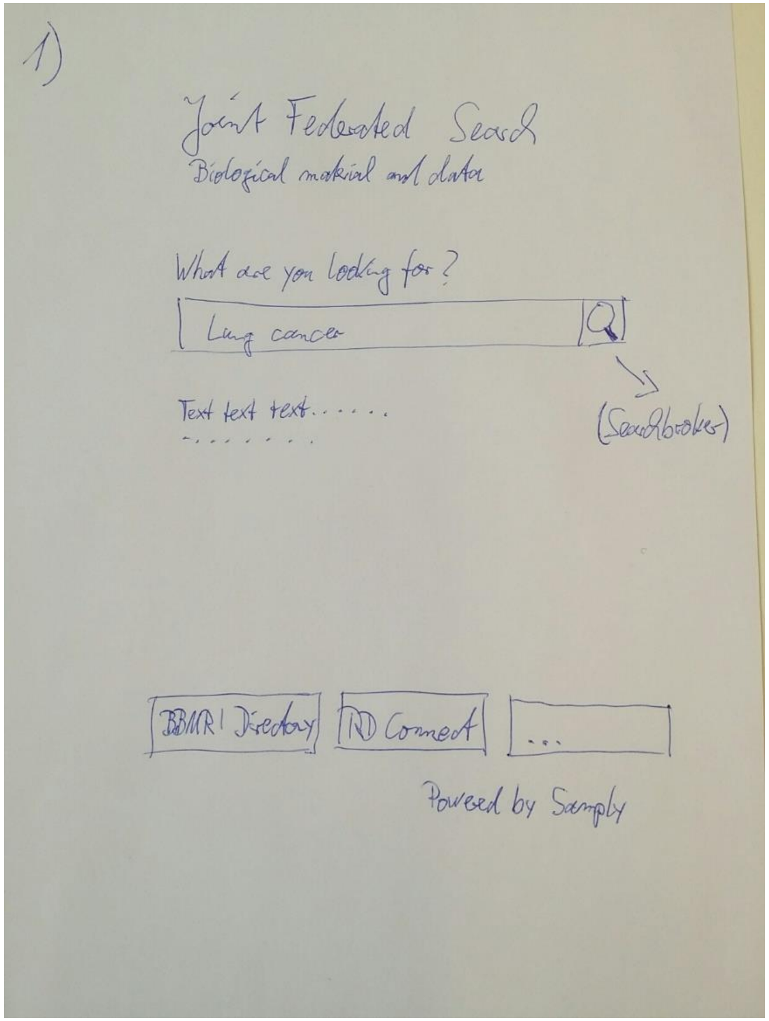

Multimedia Appendix 4. Preliminary Work: Sketched functionalities and workshop mockups.
Schüttler et al: Usability Analysis of the “Sample Locator”: a Federated Online Search Tool for Biospecimens

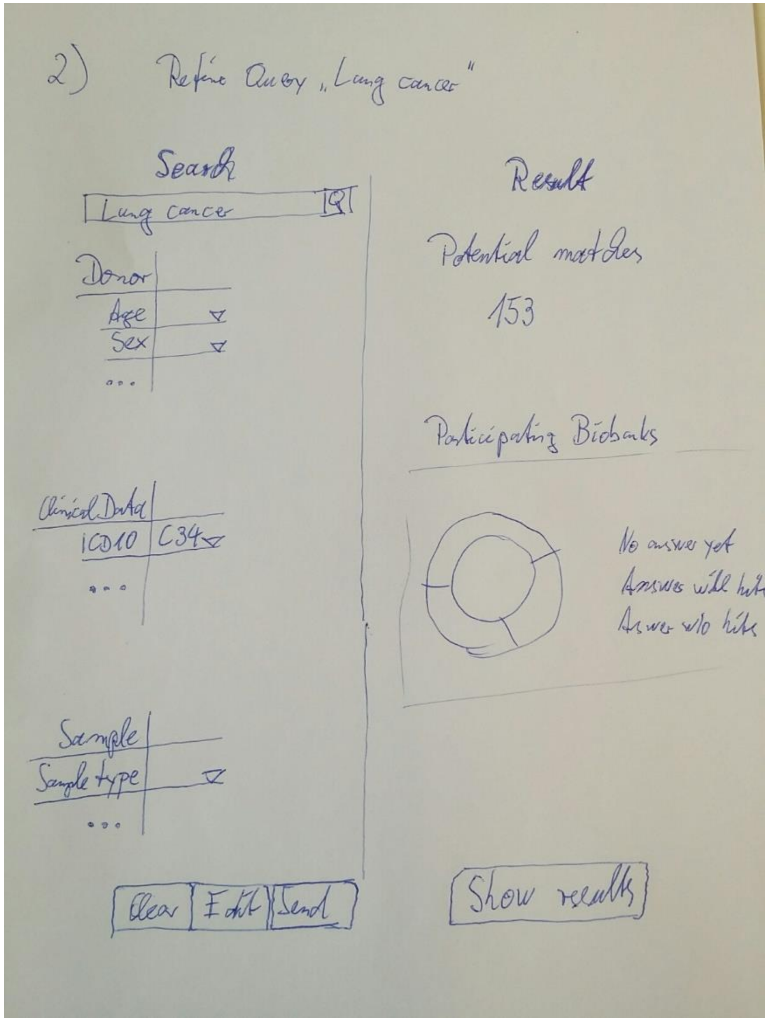

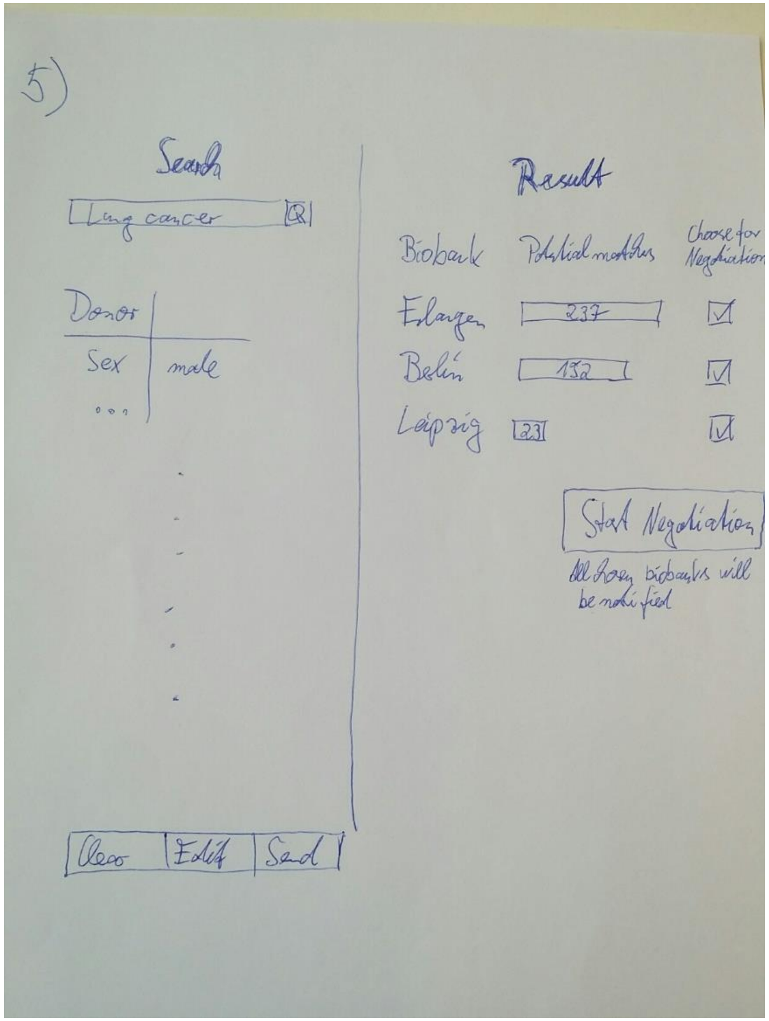
5)
Search
Lung cancer
Donor
Sex
male
Result
Biobank
Choose for Negotiation
Erlangen
237
Berlin
152
Leipzig
23
Start Negotiation
Clear
Edit
Send

B. Workshop mockups

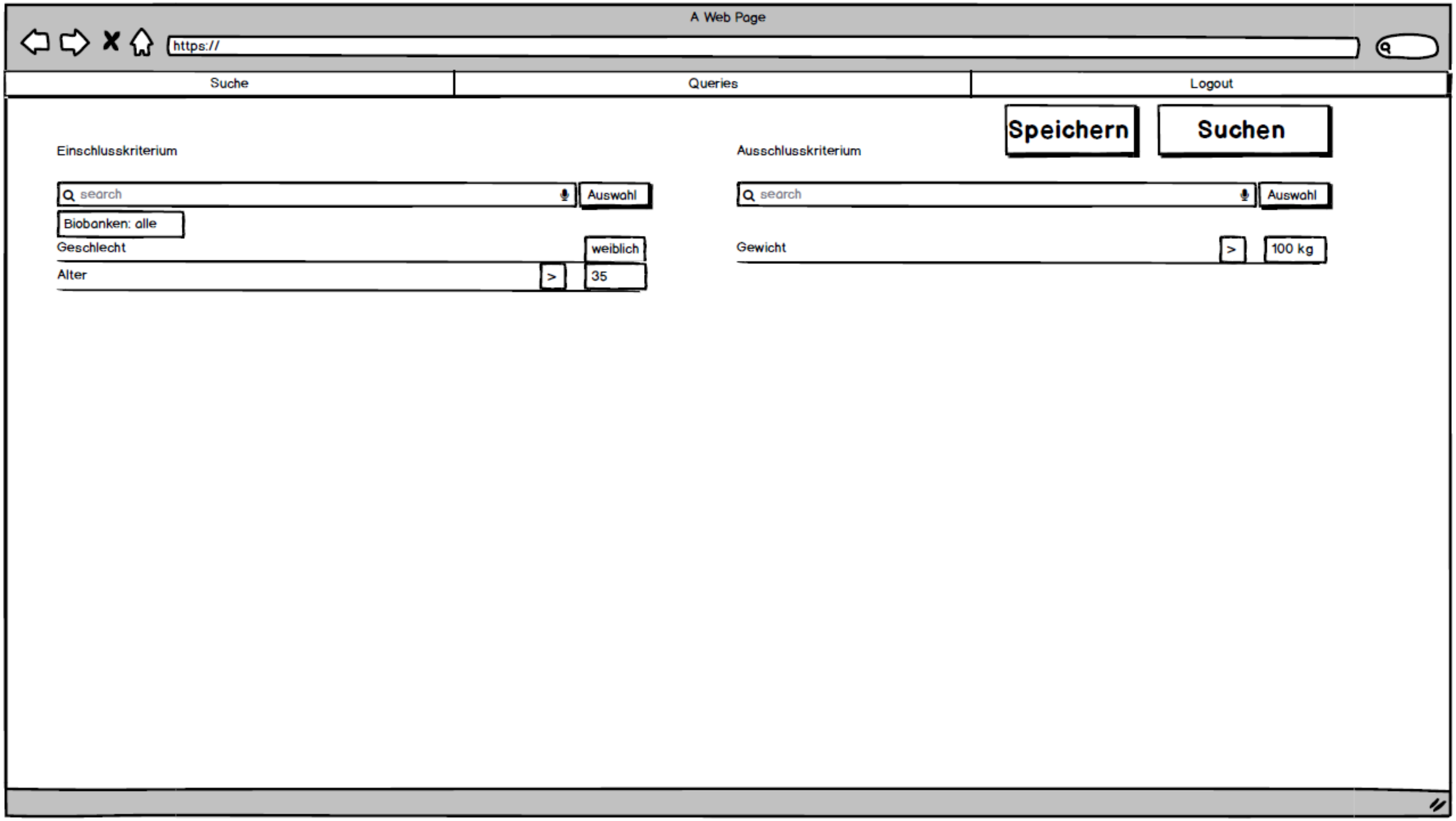

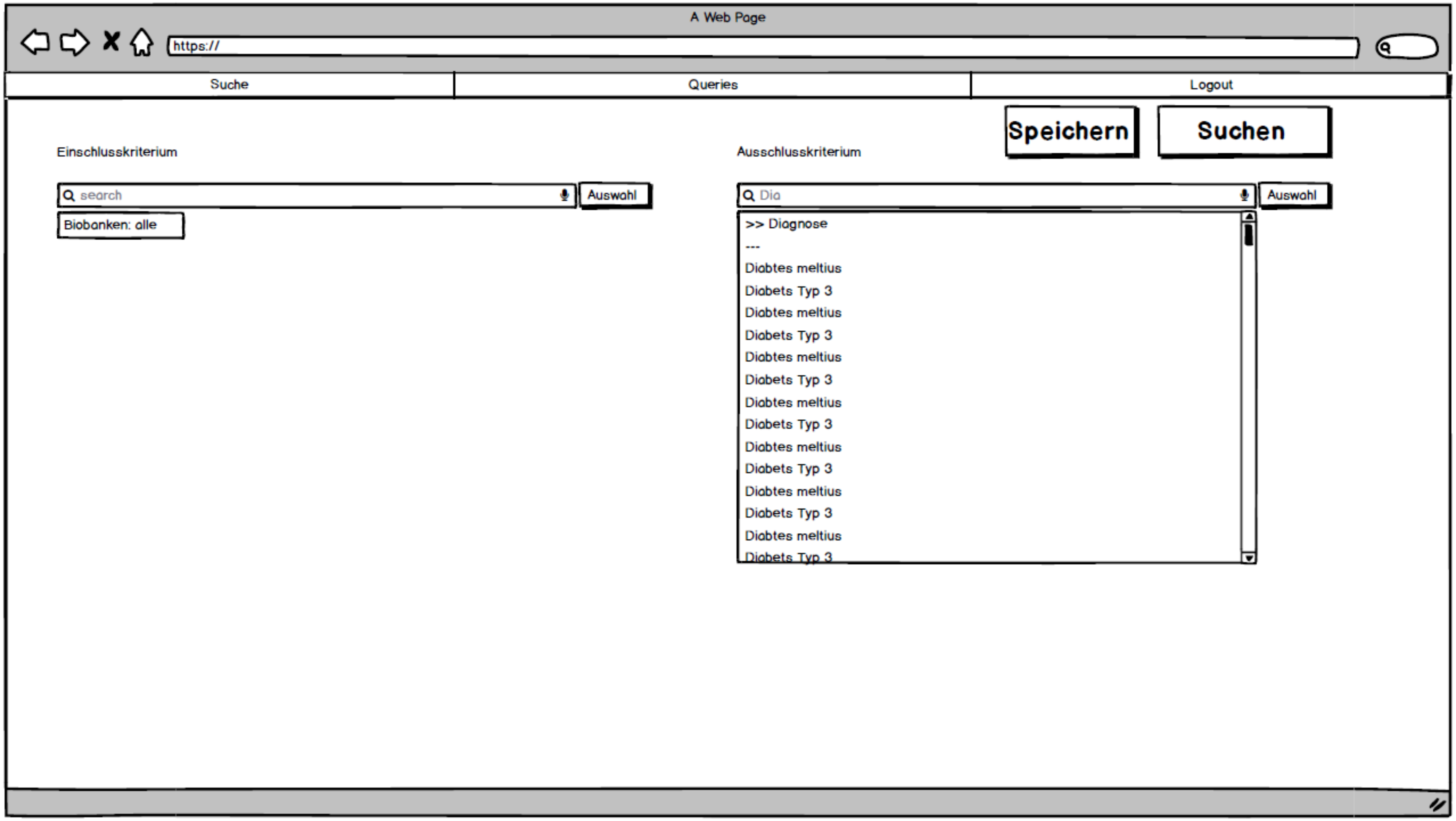
A Web Page
https://
Suche
Queries
Logout
Speichern
Suchen
Einschlusskriterium
Ausschlusskriterium
search
Auswahl
Biobanken: alle
Dia
Auswahl
>> Diagnose

Diabtes meltius
Diabets Typ 3
Diabtes meltius
Diabets Typ 3
Diabtes meltius
Diabets Typ 3
Diabtes meltius
Diabets Typ 3
Diabtes meltius
Diabets Typ 3
Diabtes meltius
Diabets Typ 3
Diabtes meltius

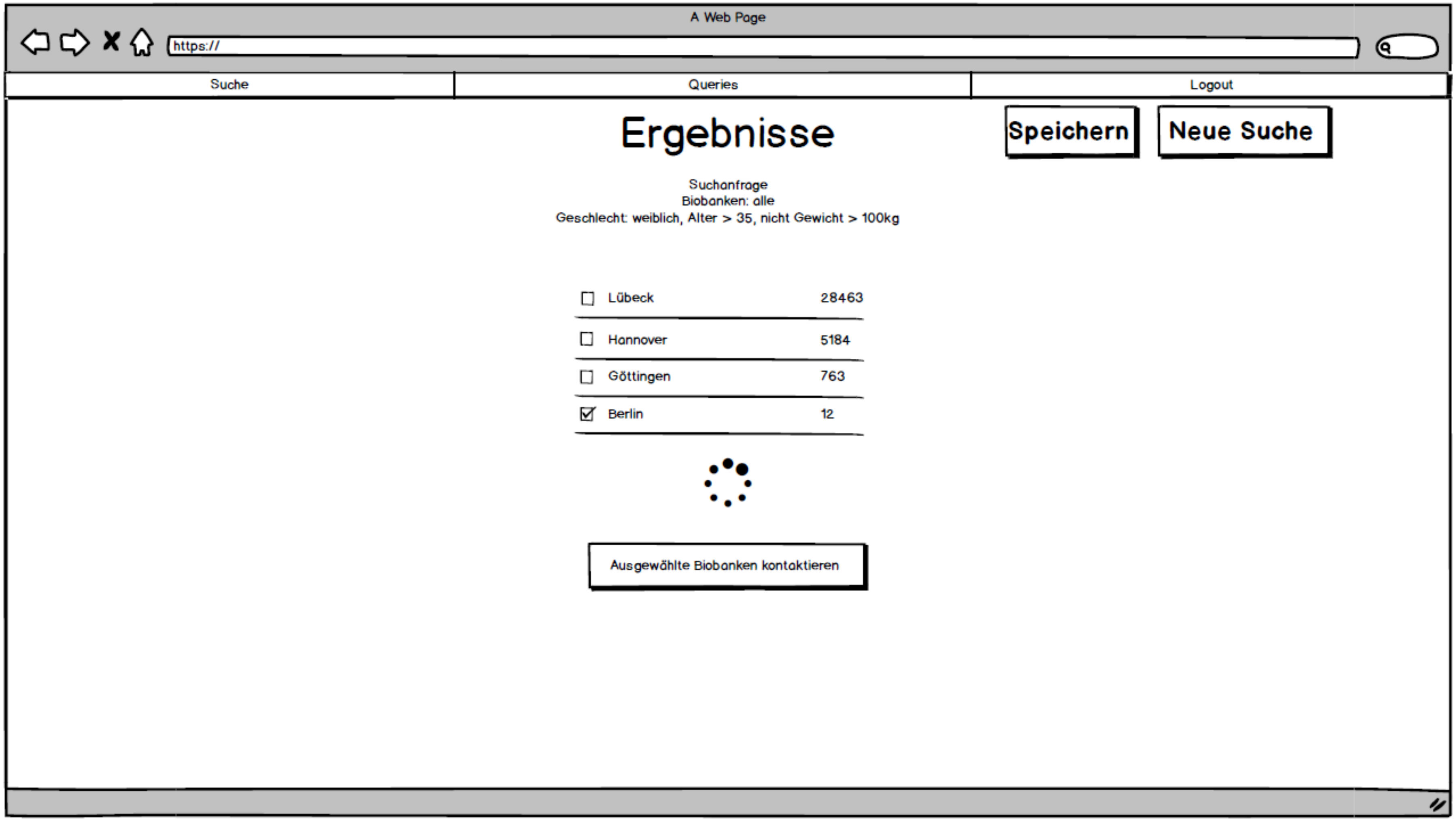
A Web Page
https://
Suche
Queries
Logout
Ergebnisse
Speichern
Neue Suche
Suchanfrage
Biobanken: alle
Geschlecht: weiblich, Alter > 35, nicht Gewicht > 100kg
Lübeck 28463
Hannover 5184
Göttingen 763
Berlin 12
Ausgewählte Biobanken kontaktieren

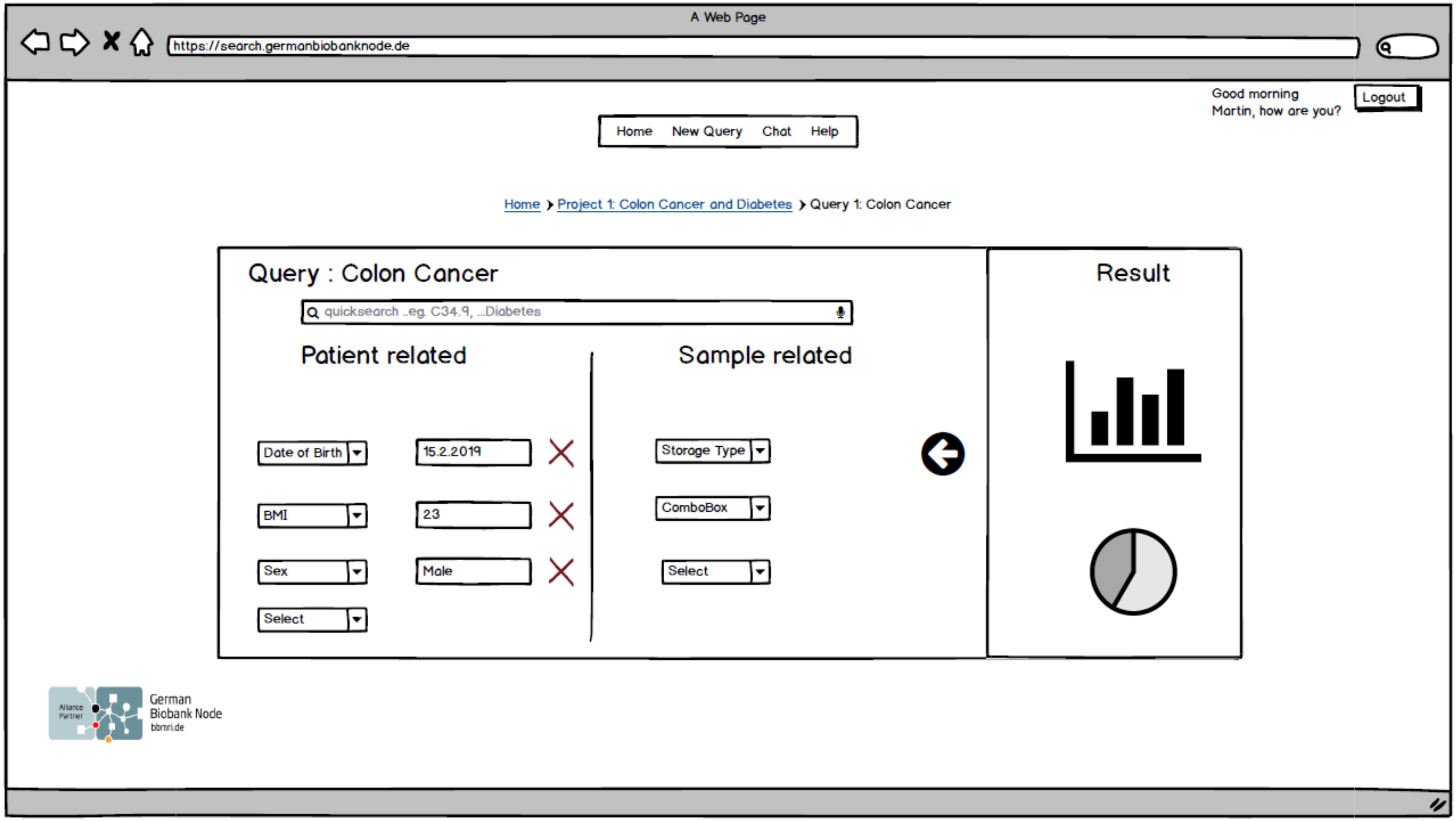
A Web Page
https://search.germanbiobanknode.de
Good morning Martin, how are you?
Logout
Home
New Query
Chat
Help
Home
Project 1: Colon Cancer and Diabetes
Query 1: Colon Cancer
Query : Colon Cancer
quicksearch ..eg. C34.9, ...Diabetes
Patient related
Sample related
Date of Birth
15.2.2019
BMI
23
Sex
Male
Select
Storage Type
ComboBox
Select
Result
German
Biobank Node
bbmri.de

Multimedia Appendix 4. Preliminary Work: Sketched functionalities and workshop mockups.
Schüttler et al: Usability Analysis of the “Sample Locator”: a Federated Online Search Tool for Biospecimens

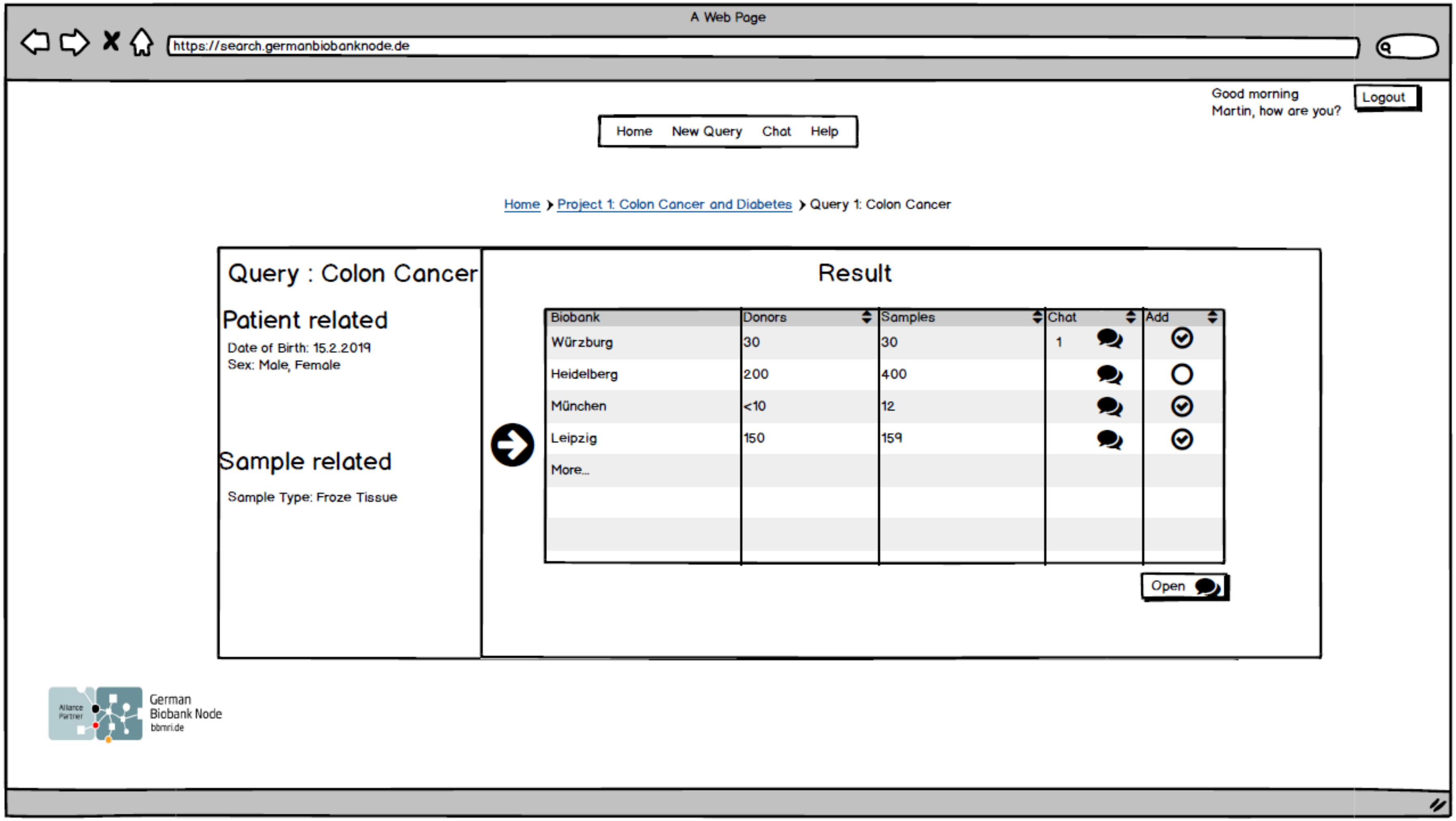

Supplement: Multimedia Appendix 4 [file jmir_v22i8e17739_app4.pdf]
