# Supplementary material for: A Federated Online Search Tool for Biospecimens (Sample Locator): Usability Study
Source: J Med Internet Res. 2020 Aug 18;22(8):e17739. doi: 10.2196/17739 (PMC7463387; doi:10.2196/17739)
Supplement: Multimedia Appendix 5 [file jmir_v22i8e17739_app5.pdf]

Multimedia Appendix 5 – Screenshots prototype

German
Biobank Node
bbmri.de

About GBN Biobanking News & Events Service Sample Locator

Home > Sample Locator DE

Help Login

Sample Locator

Search across biobanks for human biosamples and related data.

The Sample Locator allows a search for various criteria, such as sample type or ICD code.

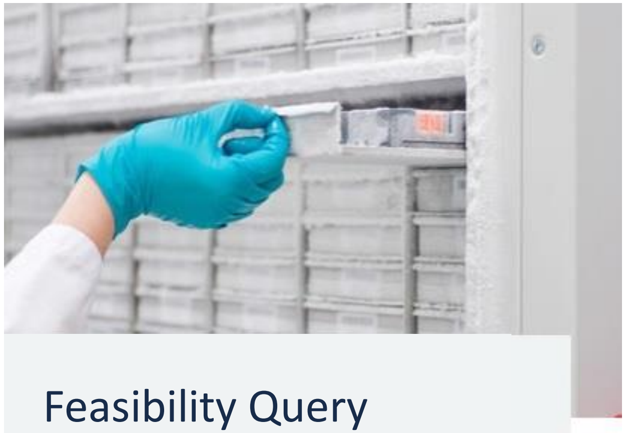

Feasibility Query
How many samples that meet your criteria are available in total?

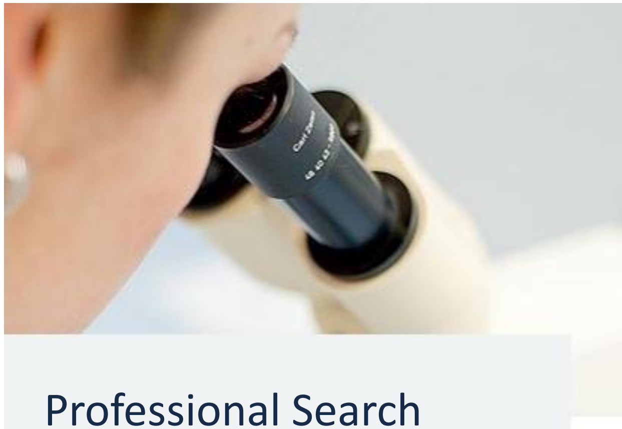

Professional Search
Log in to view sample locations and to request samples.

German Biobank Node
bbmri.de

About GBN | Biobanking | News & Events | Service | Sample Locator

Home > Sample Locator > Feasibility Query > Result

DE

Help | Login

Search	Result

Donor-related criteria ?

Sex	male
Diagnosis	C34

Sample-related criteria ?

CLEAR | EDIT

Potential matches:

**Samples**	846
**Donors**	846

To view the samples per biobank, manage your projects and search queries, and contact the biobanks, please log in.

LOGIN

© 2019 All rights reserved.

Imprint | Privacy policy

SPONSORED BY THE
Federal Ministry of Education and Research

German Biobank Node
bbmri.de

About GBN   Biobanking   News & Events   Service   Sample Locator

Home > Sample Locator > Login   DE

Help   Login

Login

Username   INPUT FIELD

Password   INPUT FIELD

BACK   LOGIN

Register   Forgot your password?

© 2019 All rights reserved.

Imprint | Privacy policy

SPONSORED BY THE
Federal Ministry of Education and Research

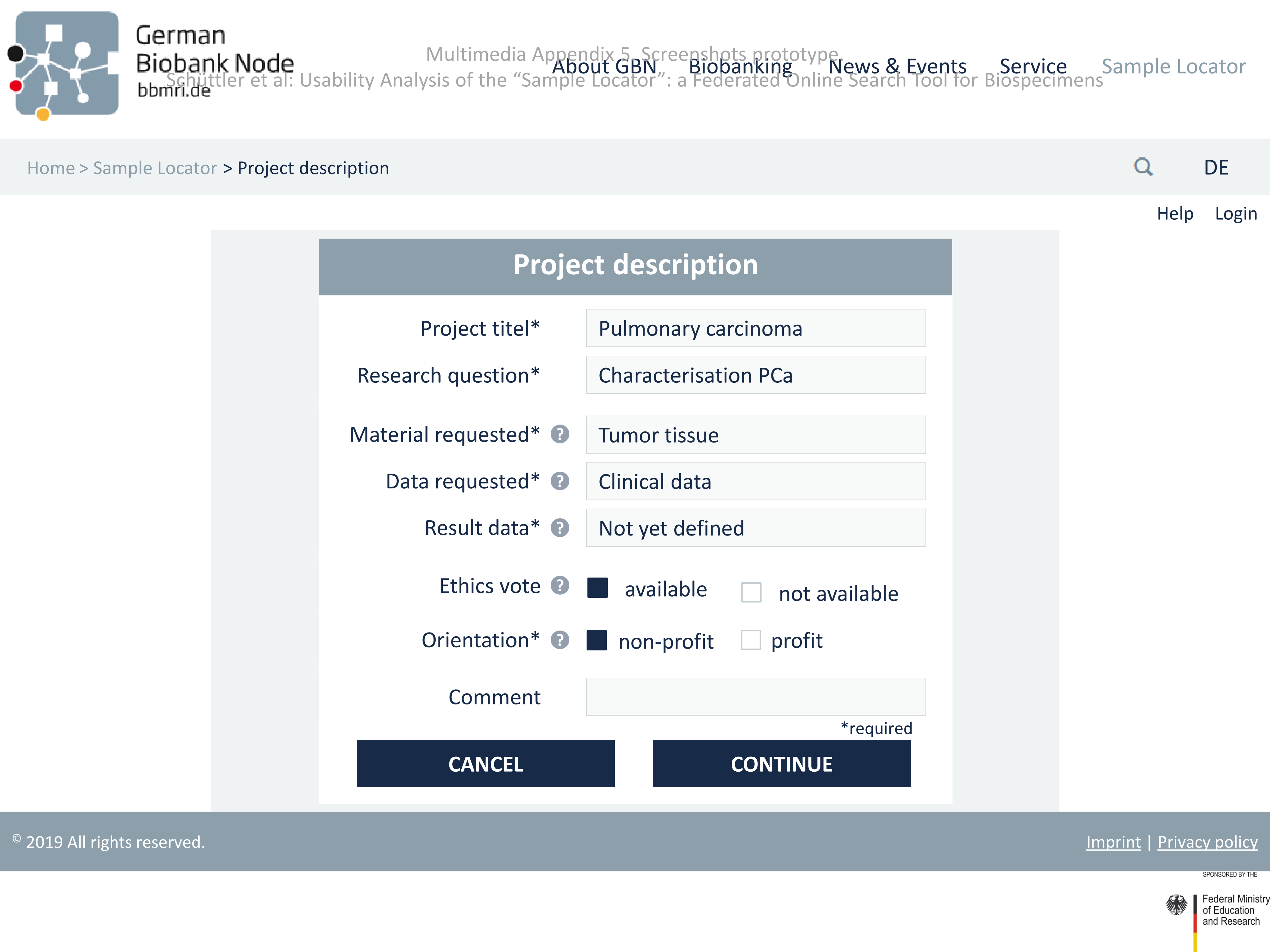
German
Biobank Node
bbmri.de
About GBN
Biobanking
News & Events
Service
Sample Locator
Home > Sample Locator > Project description
DE
Help
Login
Project description
Project titel*
Pulmonary carcinoma
Research question*
Characterisation PCa
Material requested*
Tumor tissue
Data requested*
Clinical data
Result data*
Not yet defined
Ethics vote
available
not available
Orientation*
non-profit
profit
Comment
*required
CANCEL
CONTINUE
© 2019 All rights reserved.
Imprint | Privacy policy
SPONSORED BY THE
Federal Ministry of Education and Research

German Biobank Node
bbmri.de

About GBN | Biobanking | News & Events | Service | Sample Locator

Home > Sample Locator > Query > Result

DE

Help | Max Mustermann

Search

Donor-related criteria ?

Sex	male
Diagnosis	C34

Sample-related criteria ?

CLEAR | EDIT

Result

Biobank	Samples	Donors
Lübeck	554	554
Aachen	250	250
Greifswald	22	22
Frankfurt	20	20
In total	846	846

NEGOTIATE

SAVE RESULTS

© 2019 All rights reserved.

Imprint | Privacy policy

SPONSORED BY THE
Federal Ministry of Education and Research

German Biobank Node
bbmri.de

About GBN | Biobanking | News & Events | Service | Sample Locator

Home > Sample Locator > Query > Result

DE

Help | Max Mustermann

Search

Donor-related criteria ?

	Sex	male
	Diagnosis	C34

Sample-related criteria ?

	Diagnosis	C77
OR	Diagnosis	C78
NOT	Diagnosis	C79
	Sample type tissue	Tissue formalin

CLEAR | EDIT

Result

Biobank	Samples	Donors
Lübeck	60	55
Aachen	25	22
Greifswald	10	8
Frankfurt	10	10
In total	105	95

NEGOTIATE

SAVE RESULTS

© 2019 All rights reserved.

Imprint | Privacy policy

SPONSORED BY THE
Federal Ministry of Education and Research

German Biobank Node
bbmri.de

About GBN | Biobanking | News & Events | Service | Sample Locator

Home > Sample Locator > Query > Result

DE

Help | Max Mustermann

Search

Donor-related criteria ?

Sex	male	✖
Diagnosis	C34	✖

Sample-related criteria ?

	Diagnosis	C77	✖
OR	Diagnosis	C78	✖
NOT	Diagnosis	C79	✖
	Sample type tissue	Tissue formalin	✖

CLEAR | EDIT

Result

Biobank	Samples	Donors
■ Lübeck	60	55
■ Aachen	25	22
☐ Greifswald	10	8
☐ Frankfurt	10	10
In total	105	95

NEGOTIATE

PROJECT OVERVIEW

© 2019 All rights reserved.

Imprint | Privacy policy

SPONSORED BY THE
Federal Ministry of Education and Research

German
Biobank Node
bbmri.de

About GBN Biobanking News & Events Service Sample Locator

Home > Sample Locator > Project overview DE

Help Max Mustermann

Project „Pulmonary carcinoma" EDIT

Query from 15.04.2019, 11:26 VIEW ACTIVE CHATS

ADD NEW QUERY

BACK NEW PROJECT

© 2019 All rights reserved.

Imprint | Privacy policy

SPONSORED BY THE
Federal Ministry
of Education
and Research

German
Biobank Node
bbmri.de

About GBN Biobanking News & Events Service Sample Locator

Home > Sample Locator > Query > Result > Public chat

DE

Help Max Mustermann

Projekt: „ Pulmonary carcinoma“ **Query from 15.04.2019, 11:26**

Selected Biobanks: ?

AACHEN **LÜBECK**

15.04.2019 **Max Mustermann**

I need samples from patients with lung cancer. Either frozen tissue or FFPE sections. Ideally from several pieces of the tumor.
Are such samples available at your site? 11:28

Erika Musterfrau (Biobank Lübeck)

How many samples do you need? 11:36

Markus Möglich (Biobank Aachen)

We have 25 samples, but the FFPE cuts are not from several pieces of the tumor.
11:47

Compose your message here

BACK **PROJECT OVERVIEW** **CLEAR**

© 2019 All rights reserved.

Imprint | Privacy policy

SPONSORED BY THE
Federal Ministry of Education and Research
